# Inflammation of the Skin as a Result of the Poisonous Principles of Plants

**Published:** 1897-09

**Authors:** F. H. Beadles


### INFLAMMATION OF THE SKIN AS A RESULT OF THE POISONOUS PRINCIPLES OF PLANTS.

In the study of cutaneous affections we find quite an interesting one in the inflammation of the skin caused by the influence of poisonous plants. Probably no affection of the skin is more frequently met with in the summer season. We find numerous plants, growing wild or cultivated for their beauty, which are endowed with poisonous principles, and which, by contact with or close proximity to the skin, cause inflammation of various degrees of intensity. In his monograph Dr. White mentions no less than sixty plants possessing this noxious influence.

The most frequent, and yet the severest form of dermatitis from vegetable poison is caused by toxicodendric acid, the poisonous principle of the *rhus* group, which includes:

*Rhus toxicodendron*, poison oak; *rhus diversilola*, another variety of poison oak; *rhus veneneta*, poison sumach, dogwood, or elder; *rhus radicans*, poison ivy.

The poisonous effect of these plants upon the skin depends largely upon the susceptibility of the individual. Many persons are entirely insensible to the toxic influence of plants, handling them with perfect impunity, while others are so susceptible that mere exposure to the volatile principle of the plant is sufficient to excite the most violent dermatitis.

Many cases are recorded, exemplifying this peculiar sensibility, which were caused by simply passing the growing vine or inhaling the smoke of burning wood to which the vine had clung. Besides the *rhus* group, there are a number of plants which cause inflammation only by contact with the skin, viz.: Virginia creeper, oleander, nasturtium, etc.

The agents which are employed in the treatment of this affection are numerous and varied. For a long time the solutions of lead were used almost exclusively, but, their action being slow, they have been almost entirely abandoned. The various ointments, such as oxide of zinc, tar, etc., have each had their advocates. In the treatment of numerous cases of this inflammation in my practice and in the Department of Diseases of the Skin at the City Dispensary, I have found no plan of treatment as essential and successful in the outset of the affection as the use of hot alkaline baths. These baths may be rendered alkaline with bicarbonate of sodium, biborate of sodium, or, better still, hyposulphite of sodium. The object of this bath is to neutralize the irritative, volatile principle—toxicodendric acid. After the bath the surface should be thoroughly dried and some good, soothing agent applied.

One of the most annoying symptoms in connection with this affection is the intense pruritus, for the alleviation of which many agents have been used.

Although the majority of text-books recommend cocaine for the treatment of pruritus I have found this generally valuable agent of very little service. While cocaine will invariably relieve pruritus of mucous membrane, yet it will be found disappointing in the treatment of this affection on account of not making its action felt through the intact epidermis. Still it can be used with success when the vesicles have been ruptured. An agent which I have used with great success is menthol, and in the treatment of inflammation I have obtained the happiest results from the use of the following:

Menthol ..................................gr. xv.
Fld. ext. Grindelia Robusta }
Tr. Lobelia } .....aa oz. j.
M. Sig.—Apply locally every four hours—after alkaline bath.

About a month ago I was called to treat quite an interesting case of dermatitis caused by the poison of the *nerium* (oleander). On the evening of June 20th, Miss F. L. S., aged 23, trimmed the plant and also cut off several of the blooms, the odor of which she inhaled. The following morning she found her face and hands swollen and attended with an intense throbbing, burning pain. The symptoms gradually increased in severity, until the face, hands, and arms were greatly swollen and œdematous, and the eyes almost entirely closed from the marked swelling.

In about twenty-four hours from the beginning of the attack the surface, which before was red and glazed, became studded with numerous papules and vesicles. There was at this stage the most intense pruritus, and, irritated by scratching, a great many of the vesicles became ruptured, exposing a raw, denuded surface, which finally became covered with thin, yellowish crusts.

Upon treatment the inflammatory symptoms began to subside, and in a few days all symptoms had disappeared.—F. H. BEADLES, M. D., in *Medical Register*, Richmond, Va.

